# Impedance planimetry-guided transoral mediastinal myotomy after failed submucosal tunneling for recurrent achalasia

**DOI:** 10.1055/a-2924-5746

**Published:** 2026-08-21

**Authors:** Georgios Mavrogenis, Alexandros Charalabopoulos, Dimitrios Madianos

**Affiliations:** 1 Third Space Endoscopy Unit 168211 Mediterraneo HospitalGlyfadaGreece

**Keywords:** Endoscopy Upper GI Tract, Benign strictures, POEM

Severe submucosal fibrosis remains one of the most challenging situations during peroral endoscopic myotomy (POEM). We present a rescue transoral mediastinal myotomy performed after inability to create a submucosal tunnel ( Video 1 ).

**Video 1** Video demonstration of impedance planimetry-guided transoral mediastinal myotomy.


**Fig. 1:**
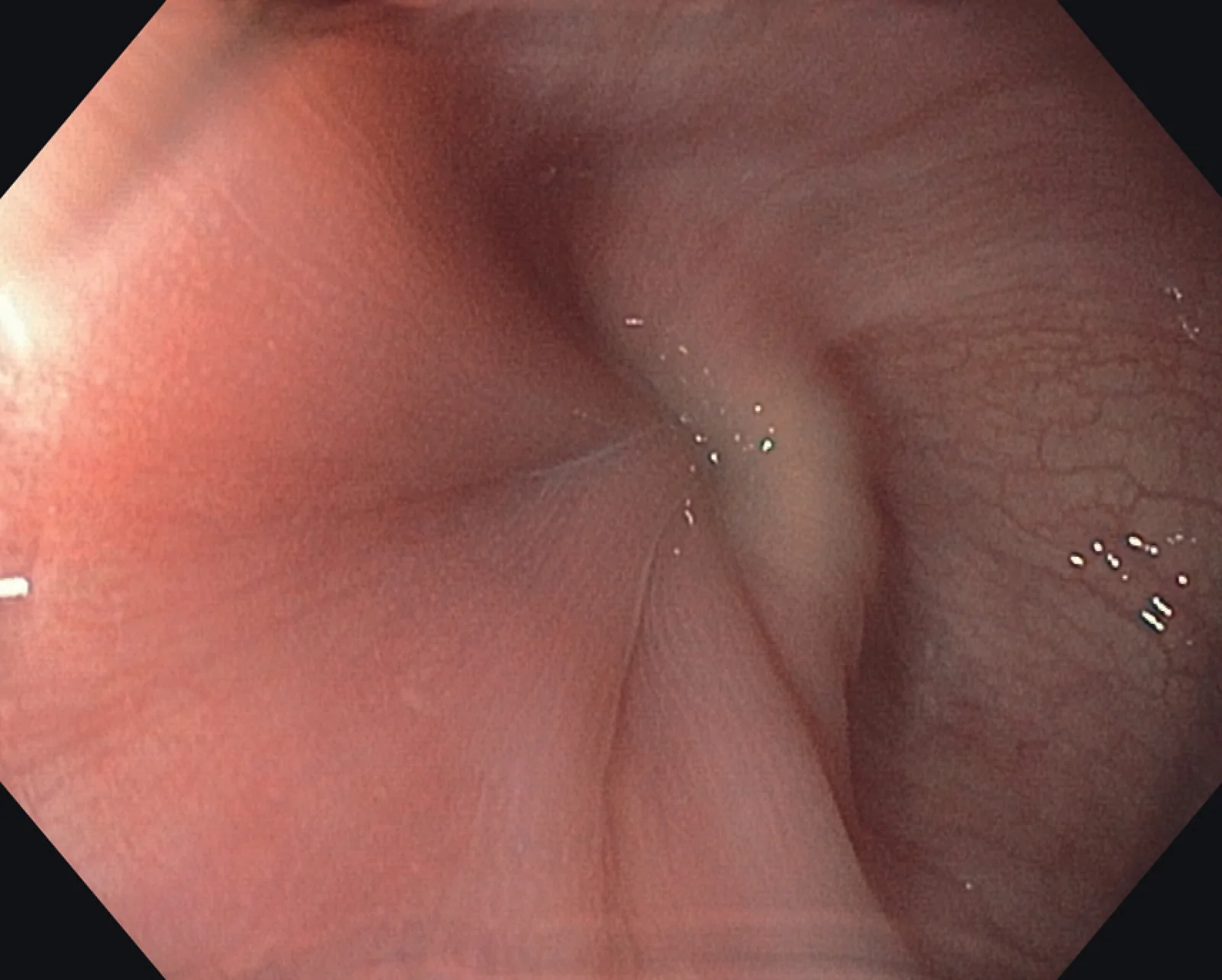
Distal esophageal stricture associated with recurrent achalasia.

**Fig. 2:**
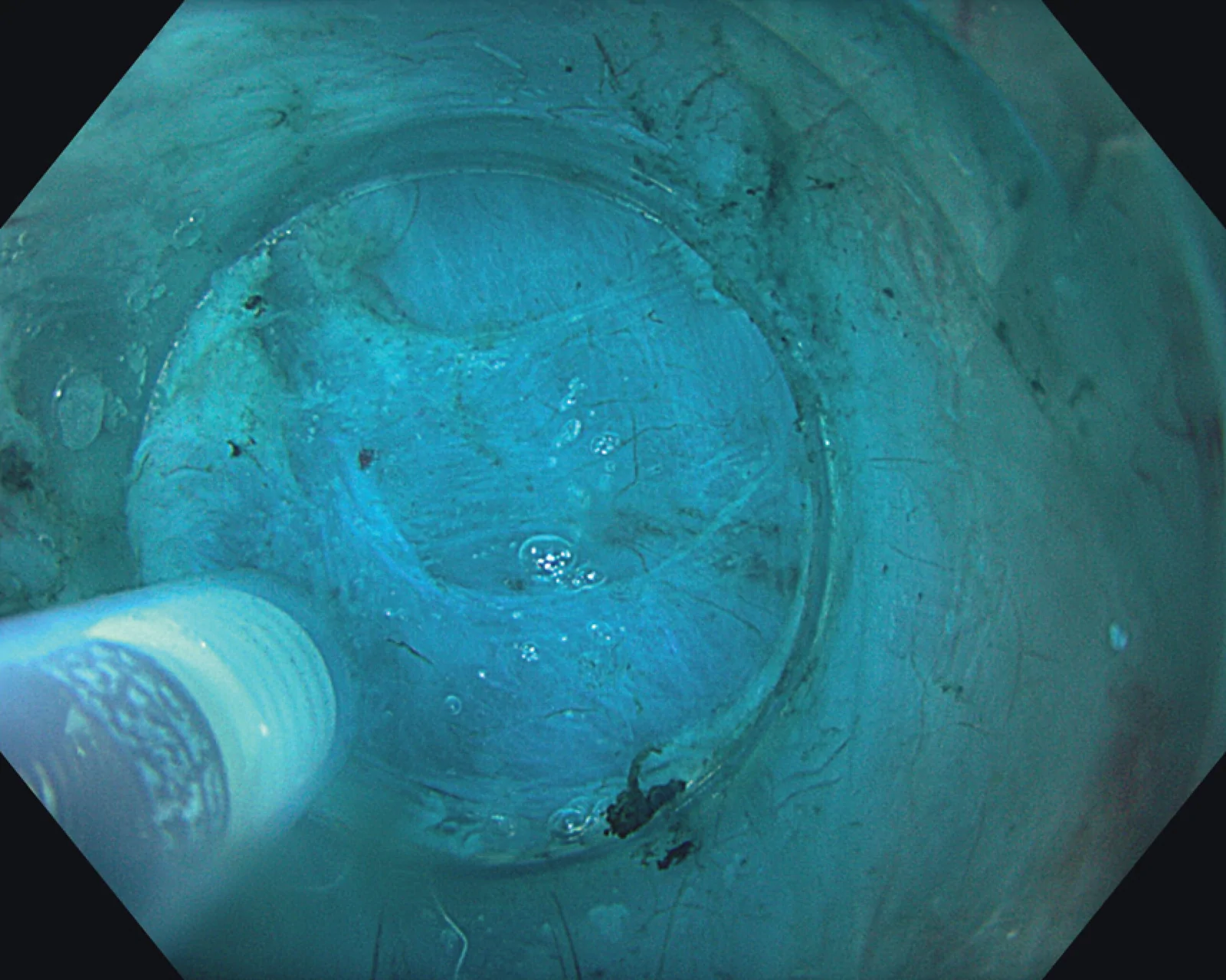
Severe submucosal fibrosis preventing the creation of a submucosal tunnel.

A 48-year-old woman presented with recurrent dysphagia and regurgitation 10 years after Heller’s myotomy ( Fig. 1 ). Posterior POEM was planned. Following mucosal incision, extensive fibrosis was encountered ( Fig. 2 ) and repeated attempts to separate the mucosa from the muscular layer were unsuccessful. Anterior tunneling was not attempted because of the previous surgical myotomy, while intramuscular dissection was deemed unfeasible. 1​

**Fig. 3:**
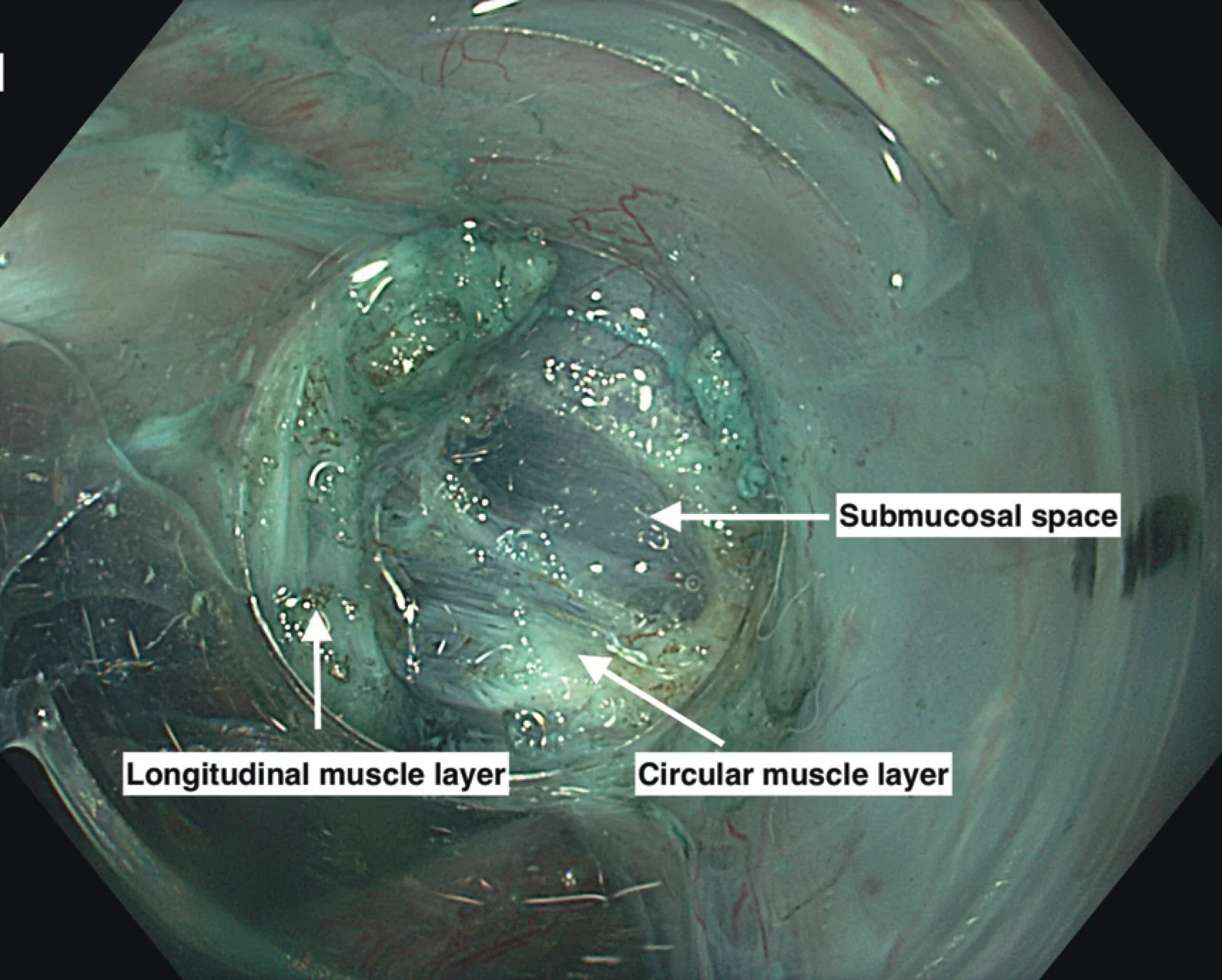
Anatomic landmarks identified during the mediastinal myotomy.

**Fig. 4:**
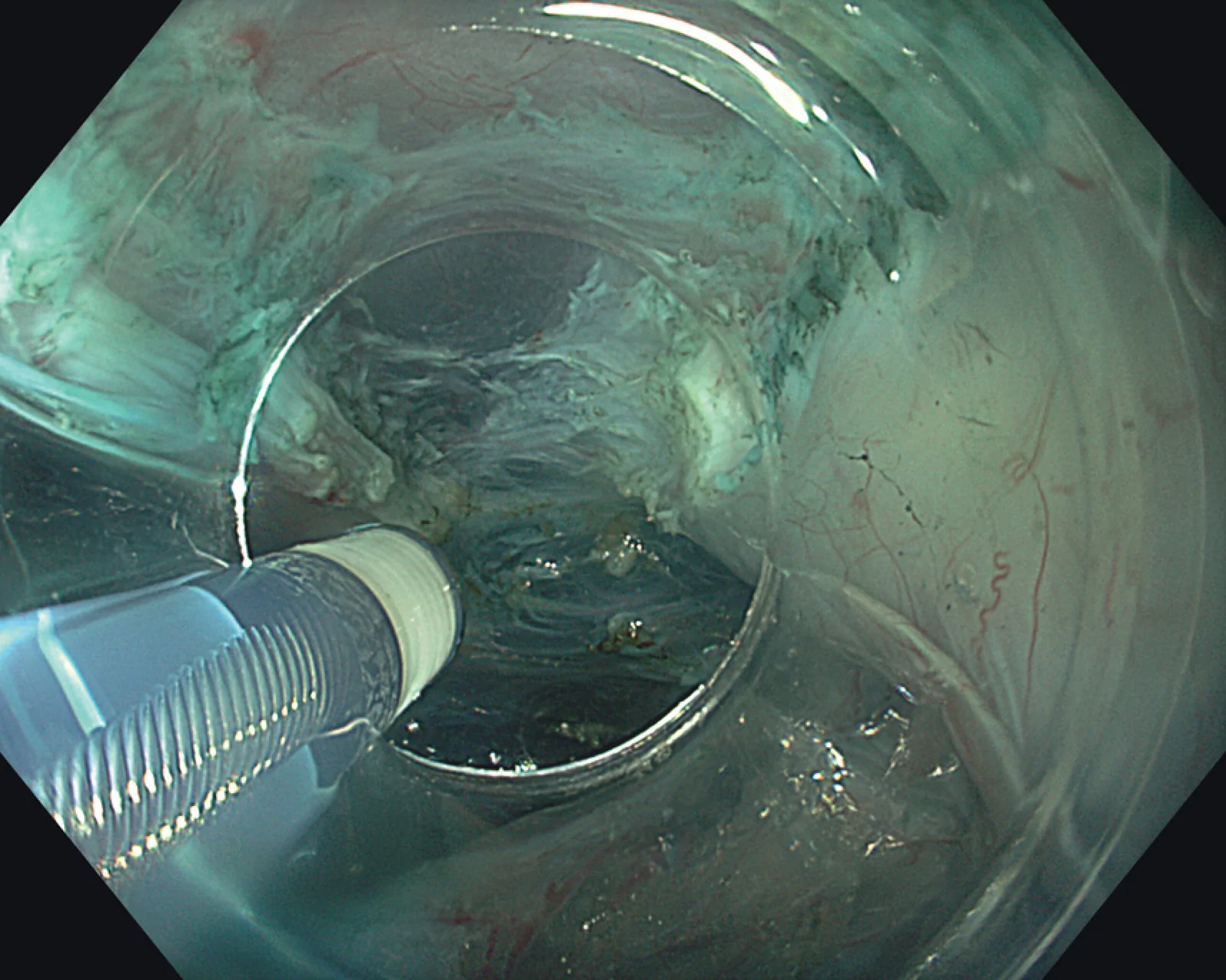
Completion of the full-thickness myotomy.

**Fig. 5:**
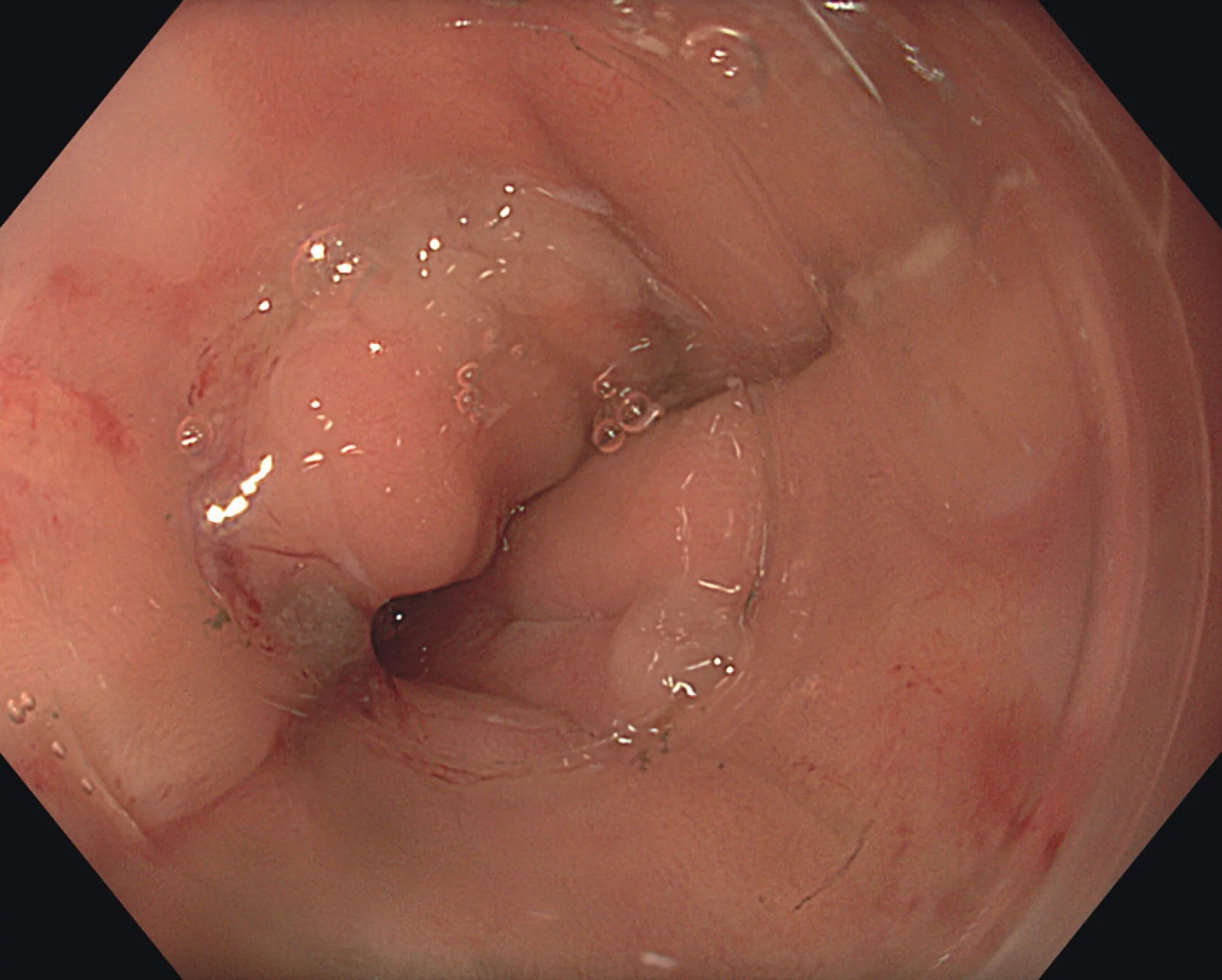
Widely patent cardia at the end of the procedure.

The endoscope was therefore advanced into the posterior mediastinum. 2​ Dissection was continued along the external surface of the esophageal wall toward the esophagogastric junction. The longitudinal muscle layer was identified and divided ( Fig. 3 ), followed by selective incision of the circular muscle fibers ( Fig.4 ), reproducing the principles of surgical Heller myotomy through a transoral endoscopic approach. Repeated submucosal injections were performed during myotomy to create a protective cushion and minimize the risk of mucosal injury. Myotomy adequacy was assessed by intermittent intraluminal inspection of cardia opening ( Fig 5 ) and by impedance-planimetry measurements, demonstrating a final distensibility index of 3.6 mm ^2^ /mm Hg. The mucosal entry was closed with clips.

Postprocedural imaging demonstrated mild pleural effusion, mild pneumothorax, and capnoperitoneum, resulting in transient chest pain that was managed conservatively. The patient was discharged the following day. At 2-month follow-up, the Eckardt score improved from 11 to 3.

This case demonstrates that transoral mediastinal myotomy may represent a feasible rescue option when severe fibrosis precludes submucosal tunneling. Intraoperative impedance-planimetry may facilitate the confirmation of myotomy adequacy in these challenging cases. Potential drawbacks of this technique include the accumulation of CO₂ and fluid within the thoracic cavity. Therefore, both CO₂ insufflation and saline immersion should be kept to a minimum whenever possible.

Endoscopy_UCTN_Code_TTT_1AO_2AP
